# An improved microwave assisted sequential extraction method followed by spectrometric analysis for metal distribution determination in South African coal samples

**DOI:** 10.1038/s41598-020-71963-2

**Published:** 2020-09-09

**Authors:** Nomvano Mketo, Philiswa N. Nomngongo

**Affiliations:** 1 Department of Chemistry, College of Science and Engineering and Technology, Florida Science Campus, University of South Africa, Roodepoort, Johannesburg, 1710 South Africa; 2 Department of Chemical Sciences, University of Johannesburg, Doornfontein, PO Box 17011, Johannesburg, 2028 South Africa

**Keywords:** Environmental sciences, Natural hazards, Chemistry, Energy science and technology

## Abstract

Some metal pollutants are corrosive in nature, are associated with fouling and slagging challenges of the coal boilers, are highly volatile and might cause air pollution and are catalyst poisoners during Fischer–Tropsch catalytic reaction. Therefore, this work describes an improved microwave-assisted sequential extraction (MW-ASE) method followed by ICP-OES/MS analysis for metal distribution determination in South African coal samples. The multivariate optimum conditions for each sequential step were 0.1 g, 200 °C and 5 min for sample amount, microwave temperature and extraction time, respectively. Under the optimum conditions, Ga, Sr and Ba were the only metals that showed solubility towards water, therefore, these metals are classified as highly mobile and eco-toxic under wet environmental conditions. Additionally, all the investigated metals showed solubility towards acidic conditions (HCl and HNO_3_). These results suggest that, most metal ions are predominantly bonded to sulphate, sulphide, and carbonate coal minerals. Alternatively, Ce, Cr and Y showed total extraction recoveries of ≤ 90%, confirming their strong affinity towards quartz coal minerals. In overall, the proposed MW-ASE method reported short extraction time (0.34 h), environmentally friendly reagents (H_2_O and diluted H_2_O_2_) and rapid multivariate optimization with acceptable extraction efficiencies (79–98%) and reproducibility (RSD ≤ 5%).

## Introduction

South Africa is one of the biggest producer of coal, which is mostly used by ESKOM for electricity generation, through combustion process and by SASOL as a raw material for Fischer–Tropsch catalytic process, for production of various petrochemicals. Additionally, high coal quality is exported to other countries for various applications. Therefore, this fossil fuel plays a crucial role in South African economy and in the energy sector. Coal is a complex mixture of hydrocarbons, non-hydrocarbons, mineral matter and Periotic Table elements, which have different physiochemical properties^1^. The large amount of coal elemental contaminants are derived from coal genesis (plants and animals), mining, processes, transportation and storage. It has to be noted that, coal elemental constituents can be associated with organic matter, mineral matter and pores of the coal, depending on their physiochemical properties. Therefore, the latter is the main determining factor for metal occurrence within the coal structure. Some of these metal contaminants are corrosive (Cr, Fe, Cl) in nature and can cause damages to the combustion boilers, thereby reducing boilers’ life span^2^. Alkali and alkaline earth metals are associated with boilers’ fouling and slagging challenges during coal burning^3^. From the environmental point of view, elements such as S, halogens, B, Hg, Cd, Se, etc. are known to be highly volatile, therefore, are expected to be released into the atmosphere and cause environmental pollution during coal processing. Additionally, sulphur species are known to be catalyst poisoners during Fischer–Tropsch catalytic reaction^1^. Therefore, development of methods that can determine coal elemental distribution is fundamental, in order to understand thermal, chemical and environmental behaviour of each element during coal conversion processes and for development of future coal clean-up strategies.

Various direct (non-destructive analytical techniques)^4^ and indirect analytical methods such as sequential chemical extraction^5^, and statistical methods^6^, have been reported for the determination of coal elemental distribution. However, sequential chemical extractions (SCE) are the commonly used methods for the determination of elemental distribution in coal related matrices^7–9^. Additionally, there were various literature reports that have been published for coal matrices as well. For example, Laban et al. developed a microwave assisted sequential extraction method using different acids (H_2_O, HCl, HNO_3_ and HNO_3_-HCl-HF) for the determination of metal association with water soluble phases, pyrite and organic matter, respectively^5^. Then the work by Zhang et al. reported the use of 1 M acetic acid, 3 M HCl, 2 M HNO_3_ and concentrated HF for sequential extraction of different metals species in coal samples^10^. In South Africa, Moyo and co-researchers investigated the use of glass column for sequential chemical extraction of water soluble and ion exchangeable elements (1 M sodium acetate); carbonates, oxides and ferric bound elements (mixture of acetic acid and oxoammonium hydrochloride); humic and fulvic bound metals (mixture of sodium hydroxide and sodium pyrophosphate); organic bound metals (mixture of nitric acid and concentrated hydrogen peroxide) and silica associated elements (mixture of hydrofluoric and perchloric acid)^11^. In 2012, Riley et al. studied the modes of occurrences for As, B, Be, Bi, Cd, Co, Cr, Cu, Hg, Mn, Mo, Ni, Pb, Sb, Th, Tl, U, Zn, S and Fe in Australian coal samples and the sequential extraction was assisted by the use of ultrasonification. Spears reported a five step sequestial extraction method for selected elements, which occurred in pore fluids and soluble minerals (mainly sulphates, but calcite is sparingly soluble), carbonates (mainly calcite), exchangeable cations and monosulphides, pyrite and carbonates (mainly dolomite and ankerite), organic matter and silicates of the coal^12^. In 2016, Yang et al. proposed an improved sequential extration method for the determination of alkali and alkaline earth metal (Na, Ca and Mg) distribution in Zhundong coals^3^. During the same year, Dević studies coal metal interactions by sequential extraction with various chemical reagents. The results showed that, Zn and Ni were highly soluble in water, therefore, these metals posed environmental concerns during wet weather conditions^13^.

Therefore, the current study aimed at developing a rapid and eco-friendly microwave based sequential extraction procedure followed by ICP-OES/MS metal determination for evaluation of heavy metal distribution and mobility in coal samples. Environmentally friendly extracting reagents such as Milli-Q water, diluted HCl (5 M), diluted HNO_3_ (2 M) and diluted H_2_O_2_-HNO_3_ (3 M)–(7 M) will be utilised. It is worthy to indicate that, to the best of our knowledge there are no studies that have reported the use of diluted hydrogen peroxide (greener reagent) for the extraction of organic bound metals. Therefore, the proposed MW-ASE is the original work. Furthermore, the efficiencies of the extraction steps will be optimised by using multivariate optimization methods. The latter have been reported to be capable to simultaneously optimize more than one parameter and evaluate interactive effects between different factors^14–23^. Lastly, during the optimization and development stages, SARM 20 will be utilised. This is because, this certified reference material contains certified known amount of the selected heavy metals under the study.

## Methods

### Reagents, materials and solutions

All chemical reagents were of high-purity and were used without any further processing. The ultrapure water with resistivity of 18 MΩ cm from a Milli-Q system (Millipore, Bedford, MA, USA) was utilised for all the preparations of calibration standards, sample solutions and rinsing of the glassware. The suprapure inorganic acids such as hydrochloric acid (30%, vv^−1^), nitric acid (65%, vv^−1^) and hydrogen peroxide (30%, vv^−1^) were purchased from Sigma-Aldrich, South Africa and were used as extracting reagents during MW-ASE method. The proposed MWASE method was validated by using three coal certified reference materials (SARMs 18, 19 and 20) and were all supplied by Council for Mineral Technology (MINTEK) in the Republic of South Africa. It has to be noted that, SARMs 20, 19 and 18 were all having an average particle size of ≤ 106 μm and were sampled from Sasolburg, Orange Free State and Witbank, respectively. In addition, the three real coal samples assigned as coal sample A (CSA), coal sample B (CSB) and coal sample C (CSC) were collected from one of the South African coal mines and were sieved to ensure that they match the same particle size as the CRMs. All glassware were pre-soaked for 24 h in diluted nitric acid (5%, vv^−1^), extensively rinsed with Milli-Q water, and dried in an oven (Digital Scientific series 2000 oven, Scientific Engineering (Pty) Ltd, South Africa) for overnight. It is worth to indicate that, the oven was also used for dehydration (50 °C) of coal samples in order to achieve constant weight amount.

Commercially available single stock solutions (1,000 mg L^−1^) of Be, Sc, V, Cr, Co, Ga, Sr, Y, Ba, Ce, Pb, and Th in 1% (vv^-1^) HNO_3_ acid were purchased from Merck, South Africa. These stock solutions were diluted in order to make three sets of external calibration standards (0.2–1, 5–80 and 100–800 μg L^−1^) to ensure that all the concentration levels of various metal ions in both samples and CRMs were accommodated. Furthermore, Indium solution of 20 µg L^−1^ was prepared from 1,000 mg L^−1^ in 1% (vv^−1^) HNO_3_, used as an internal standard for both ICP-OES and ICP-MS measurements and was also purchased from Merck, South Africa.

### Instrumentation

The proposed sequential extraction method was conducted by using the same MARS 6 One Touch Technology Microwave lab station (CEM Microwave Technology Ltd., North Caroline, USA) that was described in our research group^27^.

The sequential step 2 (5 M HCl) extracts were analysed by using Spectro ARCOS 165 ICP-OES (SPECTRO Analytical Instrument GmbH, Germany) equipped with Cetac ASX-520 auto-sampler. The other extracts from sequential steps 1 (H_2_O), 3 (2 M HNO_3_) and 4 (7 M HNO_3_-3 M H_2_O_2_) were measured for metal ions by using Perkin Elmer NexION 300 ICP-MS Spectrometer (Perkin Elmer, Waltham, MA, USA) with a triple cone interface (thereby allowing less spread of ions, photons and neutrals as were transferred to ion optics), two modes of operation (collision and standard modes) and a single quadrupole The measurement conditions of the ICP-MS were optimized daily by following supplier’s recommendations and this technique was attached with Perkin Elmer S10 autosampler. Therefore, Table 1 shows optimum operating conditions for both ICP-OES and ICPMS. It has to be noted that, argon with purity of 99.996% was purchase from Afrox, South Africa and was used for all the plasma based measurements.

**Table 1:** Operational conditions used for the determination of heavy metals by ICP OES and ICP-MS.

Parameter	ICP-MS	ICP-OES
Radio frequency generator power (W)	1,500	1,400
Auxiliary gas flow rate (L min^−1^ )	1.2	2.0
Nebulizer gas flow rate (L min^−1^ )	0.95	0.95
Plasma gas flow rate (L min^−1^)	15	13
Replicates	3	3
Spray chamber	Baffle cyclonic quartz	Double pass Scott
Nebulizer	concentric	Cross-flow
Torch configuration	–	Radial view
Analyte	Isotope (m/z)	Emission line (nm)
Be	9	313.107
Sc	45	424.683
V	51	290.880
Cr	53	267.716
Co	59	228.616
Ga	69	417.206
Sr	88	407.771
Y	89	371.030
Ba	137	233.527
Ce	140	418.660
Pb	208	220.353
Th	232	401.913

### Microwave-assisted sequential extraction (MW-ASE) procedure

The proposed MW-ASE was conducted by using the same procedure that was previously report in our research group^28^. However, the only difference is that, a mixture of 2 M HNO_3_ and 7 M H_2_O_2_ was used in the last sequential extraction step (see Table 2).

**Table 2:** Proposed microwave assisted sequential extraction steps.

Sequential step	Reagent	Target metal
1	H_2_O	Easily mobile
2	5 M HCl	Sulphate bound
3	2 M HNO_3_	Sulphide and carbonate bound
4	7 M HNO_3_-3 M H_2_O_2_	Organic bound

### Multivariate optimization for the proposed MW-ASE procedure

The multivariate optimization of extraction time (min), microwave temperature (°C) and coal amount (g) was carried out by using full factorial design and response surface method, as explained in the previous report^28^. Additionally, it is worthy to indicate that, the concentration levels of HCl, NHO_3_ and H_2_O_2_ were already optimised in the previous studies ^5,24^, therefore, these concentration levels were fixed at 5, 2 and 3 M, respectively. Lastly, the volumes of H_2_O, HCl, HNO_3_ and HNO_3_-H_2_O_2_ were fixed at 12 mL as previously reported^24–26^. It is worthy to indicate that, the minimum (−), central (0) and maximum (+) levels of the investigated factors are presented in Table 3 and the first order experimental design resulted in 11 experiments, which were later visualise by using Pareto charts. The significant factors were then further optimised by using response surface methodology (RSM), as illustrated in Table 4.

**Table 3:** Factors and levels that were used for first order 2^3^ full factorial design.

Variable	Minimum (−)	Central (0)	Maximum ( +)
CA (g) A	0.05	0.075	0.1
T (°C) B	100	150	200
Time (min) C	5	17.5	30

**Table 4:** Factors and levels used in second order central composite response surface design.

Variable	Minimum (-)	Central (0)	Maximum (+)
CA (g) A	0.03	0.05	0.11
T (°C) B	93	150	206

## Results and discussion

### First order multivariate optimization

**Figure 1:**
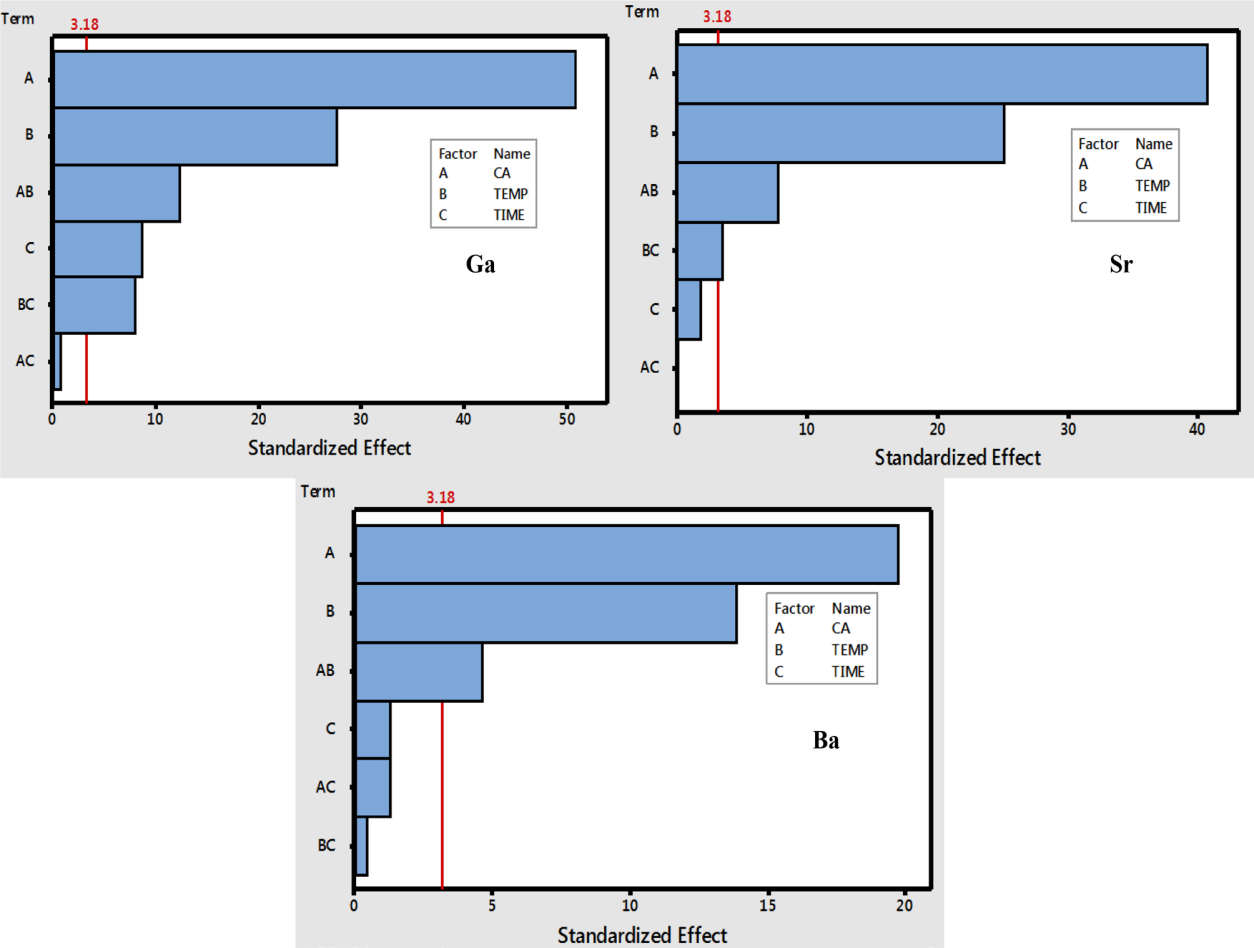
Pareto chart of the standardized effects at *p* = 0.05 for extraction of Ga, Sr and Ba using water extracting reagent.

The MW-ASE results together with design matrices of 2^3^ full factorial design for each MW-ASE reagent [H_2_O, (5 M) HCl and (2 M) HNO_3_] are shown in Tables S1–S3 (Appendix A). The investigated factors were coal amount (A), microwave temperature (B) and time (C). Additionally, these factors were examined by using analysis of variance (ANOVA) and the percent recoveries obtained for all the investigated heavy metals were used as analytical responses. The ANOVA information propagated in terms of Pareto charts was used to evaluate influential parameters and their interactions (AB, BC and AC). The charts for metal determination in water extracts are given in Fig. 1 and those of (5 M) HCl and (2 M) HNO_3_ are illustrated in Figs. S1,S2 (Appendix A), respectively. It has to be noted that, the bar length of the Pareto chart is directly proportional to the absolute value of the estimated effects. The ANOVA results observed in Fig. 1, show that the other two parameters (coal amount and temperature) exceeded the *p*-value (i.e. their bar length exceeded the red reference line) for Sr and Ba, demonstrating that coal amount and temperature are significant at 95% confidence level for the extraction of these two metals. Similar results were observed for (5 M) HCl (Fig. S1) and (2 M) HNO_3_ (Fig. S2) extracting reagents for extraction of most metals, hence time (C) was fixed at 5 min, since it was the only insignificant factor. Therefore, the significant parameters were further optimised by using RSM optimisation tool.

### Second order multivariate optimization

In order to obtain optimum conditions for the other two significant factors (microwave temperature and coal amount), second order multivariate optimization strategy was conducted, which involved the application of response surface methodology based on the central composite design. The matrix of the central composite design contained 14 experiments with responses (% recovery) correlating to each and every experimental run (see Tables S4–S6). The ANOVA data acquired and the quadratic equations (not included in the paper for simplicity reasons) of the models for each extraction reagent were used to interpret the relationship between analytical response and the evaluated parameters (microwave temperature and coal amount).

**Figure 2:**
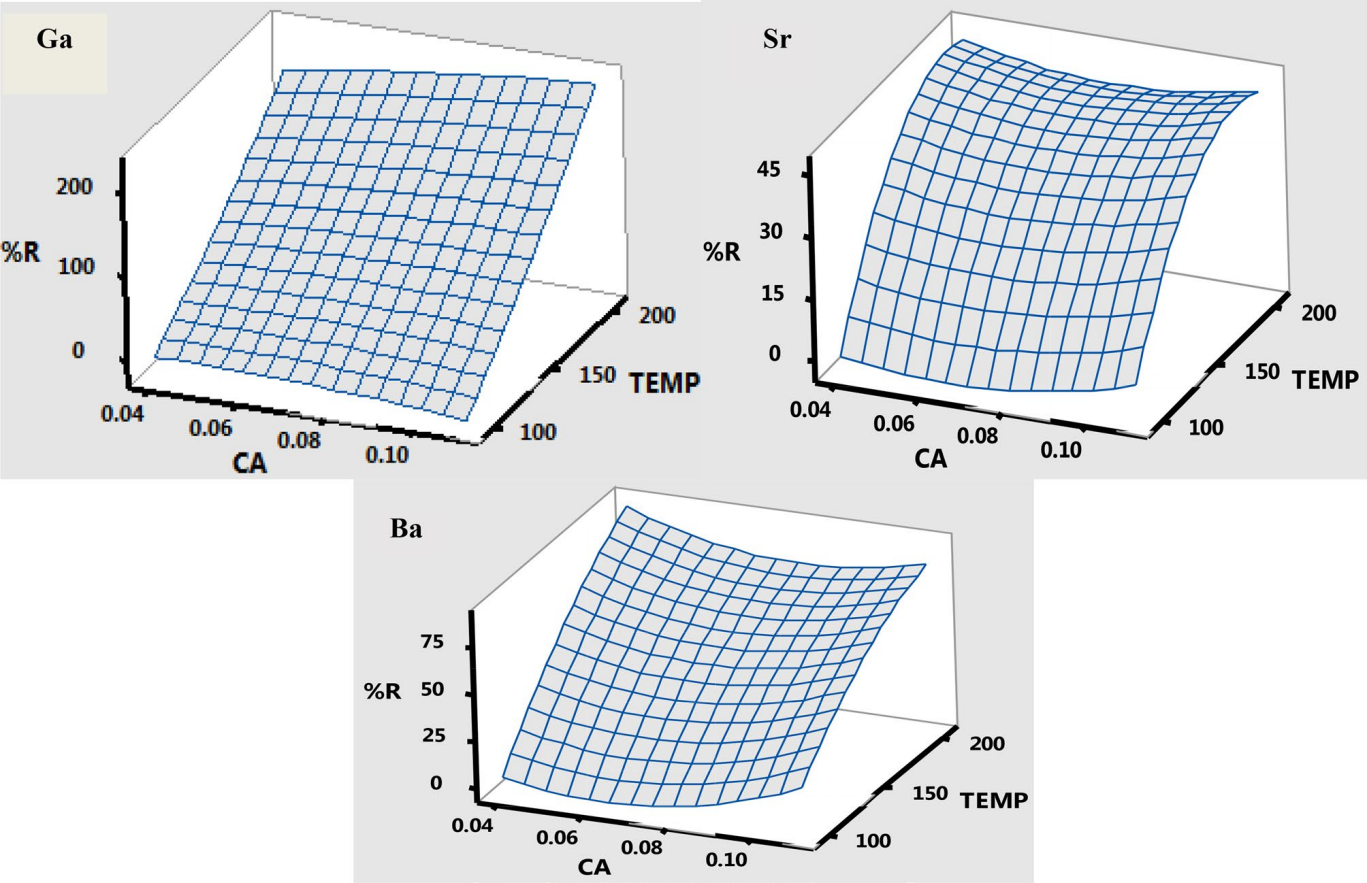
Response surface for percentage recoveries of metal ions using H_2_O extracting reagent, as a function of temperature (TEMP), °C and coal amount (CA), g at a constant extraction time of 5 min.

The three dimensional response surface plots for water extracts are illustrated in Fig. 2, the plots for 5 M HCl and 2 M HNO_3_ reagents are shown in Figs. S3,S4 (see Appendix), respectively. These three dimensional response surface plots show that, maximum analytical responses for most of the investigated metal ions (for all the examined extracting reagents) were achieved at 200 °C and 0.1 g for temperature and time, respectively. Hence, the overall interpretation of the multivariate optimization revealed that, the optimum conditions of the proposed MW-ASE were: 0.1 g sample amount, 200 °C microwave temperature and 5 min extraction time for H_2_O, HCl and HNO_3_. Additionally, the analytical responses achieved were compared with the predicted values of the RSM model and there were no significant difference at a 95% confidence levels. Therefore, the obtained optimum factors were used during validation and application stages.

### Validation of the proposed MW-ASE method

**Figure 3:**
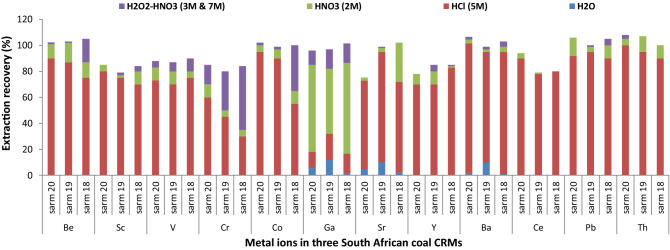
Extraction efficiencies of selected metal ions from three South Africa coal CRMs using four different extracting reagents. MW-ASE conditions: sample amount (0.1 g), microwave temperature (200 °C) and extraction time (5 min) in replicates (n = 4).

The optimum conditions of the proposed MW-ASE method were then applied in three coal CRMs (SARM 18, 19 and 20) and the results are shown in Fig. 3. The latter demonstrates that, Ga, Sr and Ba were easily extracted with pure water, therefore, these three metals can be regarded as highly mobile metal ions and can be easily introduced into the water bodies during rainy seasons. Recently, our research group reported MW-ASE method for determination of sulphur forms in coal samples and it was also observed that pure water can effectively extract ± 20% of sulphate ions in SARM 20^28^. Therefore, it can be concluded that Ba, Ga and Sr can be associated with the sulphate, carbonate and phosphate ions of the coal. Additionally, several research groups have also identified these three metal ions as easily mobile and therefore, are a threat to the environment^5,8,28^. From Fig. 3, it can also be observed that most metal ions showed strong affinity towards the HCl acidic environment, except for Ga. These observations suggest that most of metal ions are associated with the sulphate minerals of the coal and can only be mobile in acid conditions. The diluted hydrogen peroxide extracts showed minimum amount of metal ions, these results show that there are lighter interactions between elemental impurities and the organic content of the coal.

**Figure 4:**
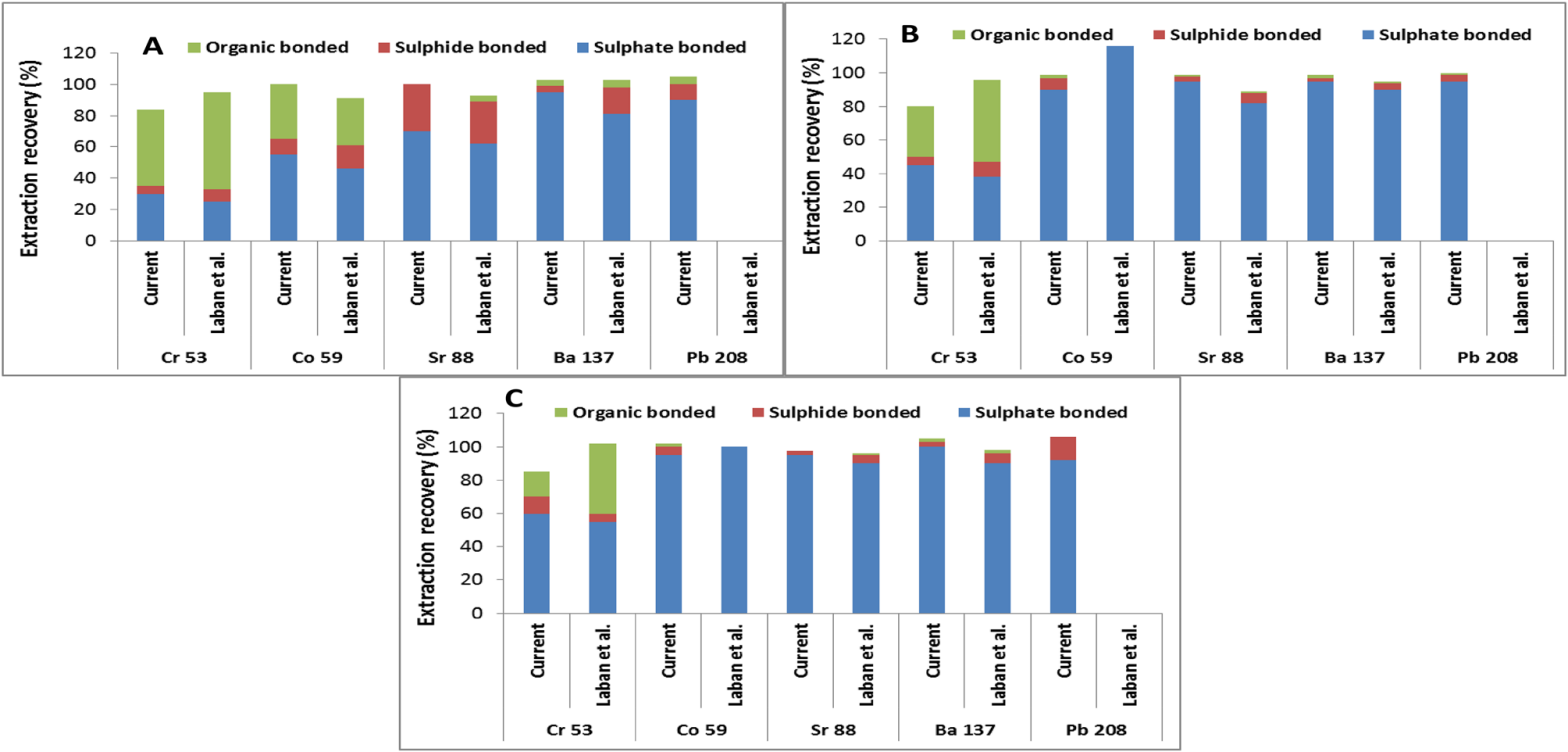
Comparison of metal extraction efficiencies of the proposed MW-ASE method and literature reported study by Laban and Atkins using South Africa coal CRMs (**A**: SARM 18, **B**: SARM 19 and **C** SARM 20).

The proposed sequential extraction was also compared with the sequential extraction method reported by Laban et al.^5^ and comparison results are illustrated in Fig. 4. The analytical results shown in Fig. 4 illustrate that the proposed the proposed MW-ASE procedure is quite comparable with the literature reported results described by Laban and Atkins for all investigated metal ions, expect for Pb. The latter showed results that were below detection limits in the literature report. This is because, the literature work used less sensitive spectrometric technique (ICP-AES) as compared to the ICP-MS used in the current study. Therefore, it can be concluded that, the proposed method is more sensitive to Pd detection as compared to Laban and Atkin’s work. However, the rest of the metal ions show similarities, especially Cr which showed some strong interaction with the organic matter of the coal in all the investigated CRMs (SARMs 18–20). It has to be noted that, even though the pattern of extraction was similar, but the proposed method showed moderate extraction recoveries (79–89%), while the literature report showed excellent extraction efficiencies (≥ 100%). The strong affinity of Cr towards organic components was also reported by several researchers^5,8,29,30^. Cobalt also reveals strong interaction with organic components of SARM 18, but was more bound with sulphate minerals in SARM 19 and 20. The rest of the metal ions were also sulphate bonded, except for strontium in SARM 18. Trace elements such as Mn and Pb are known to dominate in coal as carbonate or sulphide minerals, hence are easily solubilized in acidic medium^5,31^. The rest of the metal ions were mostly sulphate bonded, except for Sr in SARM 18. In overall, Laban and Atkin’s extraction recoveries were higher as compared to those of the current study. This is due to the kaolinite and quartz bonded minerals that could not be distracted with the chemical reagents used in the proposed MW-SAE method. It has to be noted that, HF reagent used for the destruction of the organic minerals by Laban and Atkin, can also decomposed clay minerals. The current study replaced notorious HF with environmentally friendly dilute H_2_O_2_ for the decomposition of organic matter^26,28^. Therefore, Cr partially remained in the clay part of the coal; hence cannot be quantitatively extracted without the use of hydrofluoric acid (HF)^5,8,29,32^.

### Application of the proposed MW-SAE method in real coal samples

Table 5 shows the ICP-OES/MS results that were obtained from the sequential extraction of three coal samples (CSA, CSB and CSC) received from one of the South African coal mines. From Table 5 results it can be observed that, Ga, Ba and Sr were the only metals that showed solubility toward water, this means that during wet weather conditions, these metals can be easily leached to the environment. Additionally, Ba and Sr concentration results show that, these two metals were most dominating across the whole spectrum of the three coal samples. It is also worthy to mention that, the sum concentration of most of the investigated metal ions correlated well with the concentration levels that were previous reported using total digestion methods^26,27^. This correlation confirms that, the accuracy and reliability of the proposed MW-ASE method was excellent. It worthy to indicate that, Be, Sc, Pb and Th in CSA showed slightly lower extraction efficiencies as compared to one literature report^27^. The similar trend was observed for Be and Th in CSB and for Be and V in CSC. The lower recoveries of the few metal ions (Be, Sc, V, Pb and Th) might be due to the strong affinity of these metal ions with the quartz minerals of the coal^33–35^. However, most of the challenging metal ions were not detected on the previously reported workor they were reported at lower extraction recoveries^26^. It has to be noted that, 3 mL of 7 mol^−1^ HNO_3_ was only added in order to enhance the extraction efficiencies of the investigated elements. Therefore, the proposed MW-ASE method reported 50% less consumption of HNO_3_ when compared with literature reported studies^36–38^. Therefore, the proposed MW-ASE procedure can be used as an alternative environmentally friendly method for sequential extraction of metal ions from various coal samples and related matrices.

**Table 5:** Concentration levels of trace metal distribution in three South African coal samples obtained by using the proposed MW-ASE followed by ICP-MS (n = 4) and comparison of the sequential sum values with other literature reports.

Coal sample	Extraction step	Be	Sc	V	Cr	Co	Ga	Sr	Y	Ba	Ce	Pb	Th
		Concentration (µg g^−1^)											
CSA	H_2_O	^a^ < LOD	< LOD	< LOD	< LOD	< LOD	1.21	20.0	< LOD	17.3	< LOD	< LOD	< LOD
	HCl (5 M)	1.48	2.97	29.8	9.97	3.98	5.18	350	9.56	220	35.0	4.22	4.37
	HNO_3_ (2 M)	< LOD	1.00	4.99	3.23	1.28	9.84	50.3	3.63	12.7	4.47	1.12	1.17
	H_2_O_2_-HNO_3_ (3&7 M)	< LOD	< LOD	4.89	7.34	< LOD	1.28	11.2	< LOD	3.29	< LOD	< LOD	< LOD
	^**b**^**Current study**	**1.52**	**3.96**	**40.0**	**20.5**	**5.27**	**17.5**	**412**	**13.2**	**236**	**39.5**	**5.26**	**5.58**
	**Ref. **^26^	^**c**^**ND**	**ND**	**39.4**	**ND**	**5.13**	**15.5**	**400**	**ND**	**239**	**ND**	**5.21**	**6.54**
	**Ref. **^27^	**1.91**	**4.48**	**38.5**	**20.7**	**5.19**	**16.0**	**400**	**13.5**	**236**	**40.0**	**5.56**	**5.88**
CSB	H_2_O	< LOD	< LOD	< LOD	< LOD	< LOD	1.47	11.6	< LOD	20.7	< LOD	< LOD	< LOD
	HCl (5 M)	1.77	3.68	15.9	11.4	8.18	2.97	60.5	9.29	158	37.2	17.4	8.27
	HNO_3_ (2 M)	< LOD	1.46	2.95	5.66	1.98	7.17	7.34	2.28	14.6	6.34	1.97	2.28
	H_2_O_2_-HNO_3_ (3&7 M)	< LOD	< LOD	1.18	12.0	< LOD	< LOD	3.12	< LOD	10.8	< LOD	< LOD	1.35
	**Current study**	**1.78**	**5.17**	**19.9**	**29.1**	**10.2**	**11.7**	**82.5**	**11.6**	**204**	**43.5**	**19.4**	**12.0**
	**Ref.**^26^	**ND**	**ND**	**19.4**	**ND**	**8.95**	**10.6**	**80.1**	**ND**	**189**	**ND**	**17.7**	**ND**
	**Ref.**^27^	**2.22**	**5.27**	**19.0**	**27.0**	**10.0**	**11.0**	**81.0**	**11.5**	**199**	**42.0**	**19.0**	**12.7**
CSC	H_2_O	< LOD	< LOD	< LOD	< LOD	< LOD	3.38	30.0	< LOD	73.0	< LOD	< LOD	< LOD
	HCl (5 M)	1.55	3.87	19.3	12.7	5.34	5.65	470	8.68	255	20.7	7.46	6.18
	HNO_3_ (2 M)	< LOD	1.00	7.03	6.25	2.17	12.4	25.7	1.98	21.1	11.3	2.45	1.03
	H_2_O_2_-HNO_3_ (3&7 M)	< LOD	< LOD	0.997	10.7	< LOD	1.96	15.0	< LOD	10.7	< LOD	< LOD	1.17
	**Current study**	**1.56**	**4.88**	**27.3**	**20.7**	**7.47**	**23.5**	**541**	**10.7**	**360**	**320**	**10.0**	**8.38**
	**Ref.**^26^	**ND**	**ND**	**27.3**	**ND**	**7.19**	**22.2**	**537**	**ND**	**354**	** < LOD**	**9.55**	**ND**
	**Ref.**^27^	**1.78**	**4.26**	**27.6**	**26.6**	**7.04**	**21.5**	**540**	**10.0**	**361**	**320**	**10.5**	**8.47**

^a^< LOD (below detection limits).

^b^Current study (sum of the four sequential steps).

^c^ND (not determined).

Values in bold are reported values from published literature reports.

### Comparison of current MW-ASE with literature reports

The proposed MW-ASE method was compared with other sequential extraction methods that were performed in coal related matrices for determination of various metal distribution. All the ten compared methods and the current method are illustrated in Table 6. The latter shows that, seven publications described the metal mobility in coal samples^3,5,11–13,39,41^, and the other three studies reported metal distribution in coal fly ash matrices^7,9,40^^.^ Furthermore, this table shows that, all the published literature methods reported the use of either large reagent volumes (≥ 20 mL), notorious concentrated inorganic acids or both^3,5,7,9,11–13,39,40^. However, with the proposed MW-ASE method, it was possible to use reduced diluted reagent volumes of 12 mL and environmentally friendly reagents (H_2_O and diluted H_2_O_2_). This was due to the rapid interaction of reagents and the coal, which was enhanced by the use of microwaves, as reported by Laban and Atkins^5^. Another impressive feature about the proposed methods was its short extraction time (0.34 h) as compared to the published literature reports, with extraction time ranging from 2.5 to 86 h. It has to be noted that, 2.5 h sequential extraction was also facilitated by the use of microwave system^5^. However, the only limitation about the proposed MW-ASE method was the use of high temperatures (200 °C). The latter were also reported for all the sequential extraction methods that involved the use of microwave systems^3,5,12^. The mostly investigated metal ions on various sequential extraction reports were transition metals and good recoveries (≤ 100%) were obtained. However, all the literature reported sequential extraction methods showed poor precision (≥ 10%), except for the work reported by Yang et al.^3^, Dahl et al.^9^ and the proposed MW-ASE method.

**Table 6:** Comparison of literature reported sequential extraction procedures and the proposed MW-ASE method for determination of metal occurrences in various matrices.

Sample type	Extracting agent	Extraction steps	Time (h)	Temperature (°C)	Metal ions	Recovery (%)	Precision (%)	Refs.
Coal	20 mL 0.11 M CH_3_COOH, 20 mL 0.5 M NH_2_OH.HCl & (5 mL 30% H_2_O_2_/25 mL 1 M CH_3_COONH_4_)	3	31	85	As	> 97	≤ 10	^39^
Coal	distilled water & 2 M MgCl_2_	2	16	20	Mn, Fe, Ni, Zn, Cu, Pb, Cd & Cr	NR	≤ 10	^13^
Coal	1 M NaAc, 25% HAc/0.04 M NH_2_OH.HCl, 10% NaOH/0.1 M Na_2_H_2_P_2_O_7_, 0.2 M HNO_3_/conc.H_2_O_2_ & conc. HF-HClO_4_	5	NS	60	As, Cd, Co, Cr, Mn, Ni, Pb, Th & U	NR	NR	^11^
Fly ash	20 mL H_2_O, 20 mL 1 M NaOAc, 20 mL 0.04 M NH_2_OH HCl/25% HOAc, 10 mL 30% H_2_O_2_/6 mL 0.02 M HNO_3_, 3 mL 30% H_2_O_2_ & 5 mL 3.2 M NH_4_OAc/20% HNO_3_	6	16.5	96	Cd, Cu, Fe, Mn, Pb & Zn	NR	NR	^40^
Coal	20 mL H_2_O, 20 mL 0.1 M NH_4_Cl, 20 mL 1 M HCl & conc. 9 mL HNO_3_/1 mL HF	4	3.5	210	Na, Ca & Mg	NR	≤ 7.3	^3^
Fly ash	15 mL H_2_O, 15 mL 1 M MgCl_2_, 15 mL 1 M NaOAc , 30 mL 0.04 M NH_2_OH.HCl, 6 mL HNO_3_/10 mL 30% H_2_O_2_, 10 mL 3.2 M NH_4_OAc/20% HNO_3_ & 20 mL (HNO_3_:HCl = 1:3)	7	15	25	Cd, Cr, Cu, Fe, Mn, Ni, Pb, & Zn	≥ 100	NR	^7^
Coal	200 mL H_2_O, 200 mL 1 M NH_4_OAc, 150 mL 6 M HCl, 150 mL 2 M HNO_3_ & 25 mL conc. HF/2.5 mL conc. HCl	5	74	50	As, B, Be, Bi, Cd, Co, Cr, Cu, Hg, Mn, Mo, Ni, Pb, Sb, Th, Tl, U & Zn	NR	NR	^41^
Fly ash	40 mL H_2_O, 40 mL 0.1 M CH_3_COOH, 40 mL 0.1 M NH_2_OH.HCl, 50 mL 1 M NH_4_OAc & 20 mL conc. (HNO_3_/HF/HCl)	5	86	90	Cd, Cr, Cu, Mo, Pb, Zn, As, Co, V, Ni, Ba, Al, Be, Fe, Mn, Ti, S & P	≤ 100	≤ 5	^9^
Coal	8 mL 5 M HCl/2 mL conc. HF, 10 mL 2 M HNO_3_ & conc. HNO_3_/HCl/HF	3	2.5	210	Al, Ca, Fe, Mg, P, S, Ti, Ba, Co, Cr, Cu, Mn, Ni, Pb, Sr & Zn	≥ 100	≥ 10	^5^
Coal	15 mL H_2_O, 15 mL HCl, 15 mL HNO_3_, 15 mL conc. HNO_3_ & conc. 10 HCl/5 mL HF/10 mL HNO_3_	5	4	210	34 metals	≤ 100	NR	^12^
Coal	12 mL H_2_O, 12 mL 5 M HCl, 12 mL 2 M HNO_3_ & 12 mL (7 M HNO_3_/3 M H_2_O_2_)	4	0.34	200	Be, Sc, V, Cr, Co, Ga, Sr, Y, Ba, Ce, Pb, & Th	≤ 100	≤ 5	Current study

## Conclusion

An improved microwave assisted sequential extraction (MW-ASE) method followed by ICP-OES/MS analysis was successfully developed for rapid and effective determination of metal distribution in South African coal samples. The most influential extraction parameters (coal amount, extraction time and temperature) affecting metal sequential extraction efficiencies were successfully optimised by using 2^3^ full factorial design and response surface methodology. The optimum conditions of the proposed MW-ASE were 0.1 g, 200 °C and 5 min for coal amount, microwave temperature and extraction time, respectively. Then, the optimised MW-ASE method was applied in three coal CRMs (SARM 18, 19 and 20) and three metal ions (Ga, Sr and Ba) showed solubility towards water, irrespective of the CRM. Hence, these three metals were regarded as highly mobile metal ions and are expected to leach out into the water bodies during rainy seasons. The rest of the metals were only mobile in acidic conditions, therefore, these metals can be regarded as immobile.

For validation purposes, the proposed MW-ASE method was compared with published literature work reported by Laban and Atkin using the same coal CRMs investigated in the current study. It has to be noted that, the patterns of extraction recoveries were similar for all metal ions in three different CRMs, but the proposed MW-ASE method showed moderate extraction recoveries (79–98%), while Laban’s’ work showed higher extraction recoveries (≥ 100%). The lower extraction efficiencies reported in MW-ASE method were due to the kaolinite and quartz bonded metal ions, which could not be distracted with the environmentally friendly diluted H_2_O_2_ used in the proposed procedure. However, Laban and Atkins conducted their last sequential extraction step by using notorious HF reagent, in order to also decomposed refractory clay minerals. It has to be noted that, the extraction efficiencies of the proposed MW-ASE method were also compared with extraction efficiencies that were previously reported for digestion methods and the results were quite comparable, except for few metal ions such as Be, Sc, V, Pb and Th. These metal ions were suspected to have strong affinity with the quartz minerals of the coal (see Table 5).

Furthermore, the current study was compared with other published sequential extraction methods as shown in Table 6. The latter shows some improvements when looking at the toxicity and cost. This is because; diluted environmentally friendly H_2_O_2_ was used for extraction of organic bound metals instead of concentrated notorious inorganic acids. It has to be noted that, the proposed MW-ASE method reported the use of 3 mL of 7 mol^−1^ HNO_3_, in which was only added to enhance the extraction efficiencies of the studied elements. However, HNO_3_ consumed by the proposed MW-ASE was 50% less when compared with the previously reported studies. Additionally, the proposed sequential extraction method was more accurate for direct determination of water soluble and organic bound metal ions in coal samples, in a short period of time (0.34 h) as compared to other published work (2.5 to 86 h).

## Supplementary information

Supplementary file1
