# Supplementary material for: An improved microwave assisted sequential extraction method followed by spectrometric analysis for metal distribution determination in South African coal samples
Source: Sci Rep. 2020 Sep 9;10:14841. doi: 10.1038/s41598-020-71963-2 (PMC7481181; doi:10.1038/s41598-020-71963-2)
Supplement: Supplementary file 1 — Supplementary file1 [file 41598_2020_71963_MOESM1_ESM.doc]

**Supplementary data**

**An improved microwave assisted sequential extraction method followed by spectrometric analysis for metal distribution determination in South African coal samples**

Nomvano Mketoa[[1]](#footnote-2), Philiswa N. Nomngongo^b^

*^a^Department of Chemistry, College of Science and Engineering and Technology, Florida Science Campus, University of South Africa, Roodepoort, 1710, Johannesburg, South Africa.*

*^b^Department of Chemical Sciences, University of Johannesburg, PO Box 17011, Doornfontein 2028, Johannesburg, South Africa.*

**Table S1**

Matrix of first order 2^3^ full factorial design and the analytical response (%) for each experiment and metal ions, using water as an extracting reagent

| **Run number** | **CA [A]** | **TEMP. [B]** | **TIME [C]** | **Recovery (%)** | | | | | | | | | | | |
| --- | --- | --- | --- | --- | --- | --- | --- | --- | --- | --- | --- | --- | --- | --- | --- |
|  |  |  |  | **Be** | **Sc** | **V** | **Cr** | **Co** | **Ga** | **Sr** | **Y** | **Ba** | **Ce** | **Pb** | **Th** |
| **1** | 0.05 | 100 | 5 | 0.00 | 0.00 | 0.00 | 0.00 | 0.00 | 13.5 | 9.40 | 0.00 | 3.60 | 0.00 | 0.00 | 0.00 |
| **2** | 0.1 | 100 | 5 | 0.00 | 0.00 | 0.00 | 0.00 | 0.00 | 10.3 | 7.50 | 0.00 | 2.80 | 0.00 | 0.00 | 0.00 |
| **3** | 0.05 | 200 | 5 | 0.00 | 0.00 | 0.00 | 0.00 | 0.00 | 12.8 | 8.60 | 0.00 | 3.10 | 0.00 | 0.00 | 0.00 |
| **4** | 0.1 | 200 | 5 | 0.00 | 0.00 | 0.00 | 0.00 | 0.00 | 7.80 | 5.80 | 0.00 | 1.70 | 0.00 | 0.00 | 0.00 |
| **5** | 0.05 | 100 | 30 | 0.00 | 0.00 | 0.00 | 0.00 | 0.00 | 14.8 | 9.70 | 0.00 | 3.80 | 0.00 | 0.00 | 0.00 |
| **6** | 0.1 | 100 | 30 | 0.00 | 0.00 | 0.00 | 0.00 | 0.00 | 11.7 | 7.80 | 0.00 | 2.80 | 0.00 | 0.00 | 0.00 |
| **7** | 0.05 | 200 | 30 | 0.00 | 0.00 | 0.00 | 0.00 | 0.00 | 13.0 | 8.50 | 0.00 | 3.20 | 0.00 | 0.00 | 0.00 |
| **8** | 0.1 | 200 | 30 | 0.00 | 0.00 | 0.00 | 0.00 | 0.00 | 7.70 | 5.70 | 0.00 | 1.70 | 0.00 | 0.00 | 0.00 |
| **9** | 0.075 | 150 | 17.5 | 0.00 | 0.00 | 0.00 | 0.00 | 0.00 | 15.0 | 10.0 | 0.00 | 3.60 | 0.00 | 0.00 | 0.00 |
| **10** | 0.075 | 150 | 17.5 | 0.00 | 0.00 | 0.00 | 0.00 | 0.00 | 14.9 | 10.2 | 0.00 | 3.50 | 0.00 | 0.00 | 0.00 |
| **11** | 0.075 | 150 | 17.5 | 0.00 | 0.00 | 0.00 | 0.00 | 0.00 | 15.2 | 9.80 | 0.00 | 3.80 | 0.00 | 0.00 | 0.00 |

**Table S2**

Matrix of first order 2^3^ full factorial design and the analytical response (%) for each experiment and metal ions, using HCl (5 M) as an extracting reagent

| **Run number** | **CA [A]** | **TEMP. [B]** | **TIME [C]** | **Recovery (%)** | | | | | | | | | | | |
| --- | --- | --- | --- | --- | --- | --- | --- | --- | --- | --- | --- | --- | --- | --- | --- |
|  |  |  |  | **Be** | **Sc** | **V** | **Cr** | **Co** | **Ga** | **Sr** | **Y** | **Ba** | **Ce** | **Pb** | **Th** |
| **1** | 0.05 | 100 | 5 | 0.00 | 68.4 | 42.1 | 0.00 | 65.2 | 0.00 | 62.8 | 53.9 | 31.1 | 78.9 | 19.5 | 0.00 |
| **2** | 0.1 | 100 | 5 | 22.1 | 68.4 | 44.7 | 0.00 | 66.3 | 0.00 | 72.6 | 55.3 | 58.2 | 79.1 | 0.00 | 78.2 |
| **3** | 0.05 | 200 | 5 | 0.00 | 86.1 | 68.7 | 58.0 | 73.2 | 68.0 | 67.0 | 64.7 | 38.1 | 78.6 | 96.5 | 0.00 |
| **4** | 0.1 | 200 | 5 | 46.1 | 88.0 | 78.4 | 68.2 | 82.2 | 109 | 74.8 | 65.5 | 70.6 | 81.0 | 76.7 | 83.0 |
| **5** | 0.05 | 100 | 30 | 0.00 | 74.8 | 46.6 | 0.00 | 65.1 | 0.00 | 68.7 | 54.1 | 31.1 | 76.8 | 23.0 | 0.00 |
| **6** | 0.1 | 100 | 30 | 27.4 | 72.0 | 48.7 | 0.00 | 66.4 | 0.00 | 75.3 | 55.5 | 60.8 | 79.1 | 0.00 | 78.0 |
| **7** | 0.05 | 200 | 30 | 5.20 | 91.5 | 72.4 | 67.0 | 75.3 | 111 | 69.4 | 64.5 | 39.0 | 77.9 | 101 | 0.00 |
| **8** | 0.1 | 200 | 30 | 49.7 | 89.7 | 80.9 | 69.5 | 82.2 | 105 | 74.9 | 66.0 | 70.6 | 81.0 | 90.7 | 83.5 |
| **9** | 0.075 | 150 | 17.5 | 19.9 | 78.4 | 60.4 | 45.5 | 78.1 | 30.4 | 72.1 | 59.9 | 54.3 | 78.8 | 92.6 | 83.0 |
| **10** | 0.075 | 150 | 17.5 | 20.0 | 77.9 | 60.2 | 45.3 | 78.2 | 29.5 | 71.9 | 60.1 | 54.5 | 79.0 | 91.8 | 82.9 |
| **11** | 0.075 | 150 | 17.5 | 20.1 | 78.1 | 59.9 | 45.6 | 78.0 | 30.5 | 72.5 | 60.0 | 54.7 | 79.1 | 92.1 | 83.5 |

**Table S3**

Matrix of first order 2^3^ full factorial design and the analytical response (%) for each experiment and metal ions, using HNO_3_ (2 M) as an extracting reagent

| **Run number** | **CA [A]** | **TEMP. [B]** | **TIME [C]** | **Recovery (%)** | | | | | | | | | | | |
| --- | --- | --- | --- | --- | --- | --- | --- | --- | --- | --- | --- | --- | --- | --- | --- |
|  |  |  |  | **Be** | **Sc** | **V** | **Cr** | **Co** | **Ga** | **Sr** | **Y** | **Ba** | **Ce** | **Pb** | **Th** |
| **1** | 0.05 | 100 | 5 | 58.3 | 67.4 | 55.0 | 0.00 | 60.3 | 11.7 | 62.8 | 51.6 | 47.8 | 79.4 | 0.00 | 0.00 |
| **2** | 0.1 | 100 | 5 | 58.1 | 66.7 | 53.7 | 8.40 | 68.0 | 11.7 | 72.6 | 54.4 | 62.3 | 78.0 | 16.2 | 92.3 |
| **3** | 0.05 | 200 | 5 | 78.8 | 91.4 | 73.3 | 70.9 | 90.6 | 31.2 | 67.0 | 61.7 | 57.6 | 98.7 | 0.00 | 0.00 |
| **4** | 0.1 | 200 | 5 | 78.5 | 89.8 | 70.1 | 68.5 | 91.0 | 26.4 | 74.8 | 61.8 | 74.8 | 90.6 | 75.9 | 109 |
| **5** | 0.05 | 100 | 30 | 61.8 | 72.8 | 55.3 | 0.00 | 60.4 | 11.7 | 68.7 | 51.6 | 46.5 | 79.4 | 0.00 | 0.00 |
| **6** | 0.1 | 100 | 30 | 62.4 | 72.1 | 53.1 | 8.50 | 70.0 | 11.8 | 75.3 | 54.8 | 63.8 | 78.2 | 25.0 | 92.4 |
| **7** | 0.05 | 200 | 30 | 80.0 | 90.6 | 73.1 | 70.4 | 90.8 | 30.5 | 69.4 | 61.6 | 56.8 | 98.4 | 0.00 | 0.00 |
| **8** | 0.1 | 200 | 30 | 79.6 | 88.7 | 70.8 | 69.0 | 90.6 | 28.9 | 74.9 | 61.6 | 73.7 | 90.8 | 92.0 | 98.7 |
| **9** | 0.075 | 150 | 17.5 | 73.7 | 85.6 | 65.4 | 49.2 | 83.0 | 21.9 | 72.0 | 61.0 | 63.8 | 92.9 | 72.0 | 90.7 |
| **10** | 0.075 | 150 | 17.5 | 74.0 | 86.0 | 65.5 | 49.1 | 82.9 | 22.0 | 71.9 | 60.9 | 64.0 | 93.0 | 72.5 | 90.5 |
| **11** | 0.075 | 150 | 17.5 | 73.9 | 85.9 | 65.2 | 49.0 | 83.1 | 22.1 | 72.1 | 61.1 | 63.9 | 93.1 | 71.9 | 90.0 |

**Figure S1**: Pareto chart of the standardized effects at *p* = 0.05 for extraction of the selected heavy metals using (5 M) HCl extracting reagent.

**Figure S2**: Pareto chart of the standardized effects at *p* = 0.05 for extraction of the selected heavy metals using (2 M) HNO_3_ extracting reagent.

**Table S4**

Matrix of second order 2^3^ full factorial design and the analytical response (%) for each experiment and metal ions, using water as an extracting reagent

| **Run number** | **CA [A]** | **TEMP. [B]** | **Recovery (%)** | | | | | | | | | | | |
| --- | --- | --- | --- | --- | --- | --- | --- | --- | --- | --- | --- | --- | --- | --- |
|  |  |  | **Be** | **Sc** | **V** | **Cr** | **Co** | **Ga** | **Sr** | **Y** | **Ba** | **Ce** | **Pb** | **Th** |
| **1** | 0.05 | 110 | 0.00 | 0.00 | 0.00 | 0.00 | 0.00 | 16.5 | 10.0 | 0.00 | 13.4 | 0.00 | 0.00 | 0.00 |
| **2** | 0.1 | 110 | 0.00 | 0.00 | 0.00 | 0.00 | 0.00 | 11.8 | 12.8 | 0.00 | 18.2 | 0.00 | 0.00 | 0.00 |
| **3** | 0.05 | 190 | 0.00 | 0.00 | 0.00 | 0.00 | 0.00 | 163 | 42.4 | 0.00 | 75.5 | 0.00 | 0.00 | 0.00 |
| **4** | 0.1 | 190 | 0.00 | 0.00 | 0.00 | 0.00 | 0.00 | 188 | 42.5 | 0.00 | 72.0 | 0.00 | 0.00 | 0.00 |
| **5** | 0.075 | 150 | 0.00 | 0.00 | 0.00 | 0.00 | 0.00 | 93.0 | 35.0 | 0.00 | 45.0 | 0.00 | 0.00 | 0.00 |
| **6** | 0.075 | 150 | 0.00 | 0.00 | 0.00 | 0.00 | 0.00 | 93.1 | 34.9 | 0.00 | 44.9 | 0.00 | 0.00 | 0.00 |
| **7** | 0.075 | 150 | 0.00 | 0.00 | 0.00 | 0.00 | 0.00 | 92.9 | 35.1 | 0.00 | 45.1 | 0.00 | 0.00 | 0.00 |
| **8** | 0.04 | 150 | 0.00 | 0.00 | 0.00 | 0.00 | 0.00 | 83.5 | 40.9 | 0.00 | 57.1 | 0.00 | 0.00 | 0.00 |
| **9** | 0.11 | 150 | 0.00 | 0.00 | 0.00 | 0.00 | 0.00 | 95.4 | 42.5 | 0.00 | 60.9 | 0.00 | 0.00 | 0.00 |
| **10** | 0.075 | 93 | 0.00 | 0.00 | 0.00 | 0.00 | 0.00 | 14.8 | 3.80 | 0.00 | 1.30 | 0.00 | 0.00 | 0.00 |
| **11** | 0.075 | 205 | 0.00 | 0.00 | 0.00 | 0.00 | 0.00 | 154 | 42.3 | 0.00 | 70.1 | 0.00 | 0.00 | 0.00 |
| **12** | 0.075 | 150 | 0.00 | 0.00 | 0.00 | 0.00 | 0.00 | 93.1 | 35.1 | 0.00 | 45.1 | 0.00 | 0.00 | 0.00 |
| **13** | 0.075 | 150 | 0.00 | 0.00 | 0.00 | 0.00 | 0.00 | 93.0 | 34.8 | 0.00 | 45.2 | 0.00 | 0.00 | 0.00 |
| **14** | 0.075 | 150 | 0.00 | 0.00 | 0.00 | 0.00 | 0.00 | 92.8 | 34.9 | 0.00 | 44.9 | 0.00 | 0.00 | 0.00 |

**Table S5**

Matrix of second order 2^3^ full factorial design and the analytical response (%) for each experiment and metal ions, using (5 M) HCl as an extracting reagent

| **Run number** | **CA [A]** | **TEMP. [B]** | **Recovery (%)** | | | | | | | | | | | |
| --- | --- | --- | --- | --- | --- | --- | --- | --- | --- | --- | --- | --- | --- | --- |
|  |  |  | **Be** | **Sc** | **V** | **Cr** | **Co** | **Ga** | **Sr** | **Y** | **Ba** | **Ce** | **Pb** | **Th** |
| **1** | 0.05 | 110 | 41.1 | 64.3 | 49.1 | 11.2 | 71.1 | 0.00 | 75.2 | 55.3 | 29.3 | 72.2 | 173 | 76.1 |
| **2** | 0.1 | 110 | 52.4 | 66.2 | 47.8 | 12.6 | 71.1 | 0.00 | 76.6 | 55.0 | 54.5 | 78.5 | 133 | 75.5 |
| **3** | 0.05 | 190 | 56.7 | 80.6 | 70.4 | 51.6 | 83.6 | 55.7 | 75.0 | 62.6 | 31.8 | 79.9 | 192 | 83.1 |
| **4** | 0.1 | 190 | 66.5 | 79.2 | 69.3 | 58.3 | 80.3 | 64.3 | 78.9 | 61.7 | 63.7 | 78.5 | 171 | 85.2 |
| **5** | 0.075 | 150 | 53.0 | 72.9 | 58.9 | 33.9 | 69.9 | 20.9 | 76.9 | 58.9 | 47.9 | 76.9 | 174 | 67.9 |
| **6** | 0.075 | 150 | 52.9 | 73.0 | 59.0 | 34.0 | 69.8 | 21.1 | 77.0 | 59.0 | 48.0 | 77.0 | 174 | 68.0 |
| **7** | 0.075 | 150 | 53.1 | 73.1 | 59.1 | 34.1 | 70.1 | 21.0 | 77.1 | 59.2 | 48.1 | 77.1 | 175 | 68.1 |
| **8** | 0.04 | 150 | 40.8 | 72.2 | 60.1 | 29.6 | 78.9 | 19.0 | 74.1 | 59.4 | 16.8 | 76.6 | 198 | 76.6 |
| **9** | 0.11 | 150 | 60.1 | 73.2 | 58.0 | 35.5 | 75.6 | 20.7 | 77.5 | 58.5 | 62.5 | 81.2 | 161 | 80.1 |
| **10** | 0.075 | 93 | 46.5 | 63.9 | 48.1 | 6.90 | 69.9 | 0.00 | 74.0 | 54.6 | 43.5 | 73.4 | 144 | 75.9 |
| **11** | 0.075 | 205 | 64.5 | 81.5 | 71.8 | 62.4 | 83.3 | 79.2 | 75.5 | 62.4 | 52.9 | 78.2 | 187 | 84.4 |
| **12** | 0.075 | 150 | 53.2 | 73.1 | 59.1 | 33.8 | 69.9 | 20.8 | 76.8 | 59.1 | 47.8 | 77.1 | 174 | 67.8 |
| **13** | 0.075 | 150 | 52.8 | 73.0 | 58.8 | 34.2 | 70.2 | 21.2 | 77.0 | 58.8 | 48.0 | 76.8 | 175 | 67.9 |
| **14** | 0.075 | 150 | 53.0 | 72.8 | 59.0 | 34.0 | 70.0 | 21.0 | 77.2 | 59.0 | 48.1 | 77.0 | 175 | 68.0 |

**Table S6**

Matrix of second order 2^3^ full factorial design and the analytical response (%) for each experiment and metal ions, using (2 M) HNO_3_ as an extracting reagent

| **Run number** | **CA [A]** | **TEMP. [B]** | **Recovery (%)** | | | | | | | | | | | |
| --- | --- | --- | --- | --- | --- | --- | --- | --- | --- | --- | --- | --- | --- | --- |
|  |  |  | **Be** | **Sc** | **V** | **Cr** | **Co** | **Ga** | **Sr** | **Y** | **Ba** | **Ce** | **Pb** | **Th** |
| **1** | 0.05 | 110 | 60.0 | 68.0 | 50.3 | 0.00 | 78.0 | 0.00 | 67.7 | 53.6 | 17.2 | 80.2 | 0.00 | 73.7 |
| **2** | 0.1 | 110 | 60.0 | 68.1 | 51.6 | 6.50 | 74.1 | 4.80 | 75.8 | 53.4 | 44.5 | 78.3 | 83.3 | 85.8 |
| **3** | 0.05 | 190 | 75.2 | 87.2 | 64.2 | 54.7 | 89.2 | 12.3 | 75.7 | 62.1 | 21.2 | 91.3 | 29.4 | 103 |
| **4** | 0.1 | 190 | 75.8 | 86.6 | 65.9 | 64.2 | 86.8 | 17.2 | 83.1 | 60.6 | 54.0 | 85.2 | 128 | 93.9 |
| **5** | 0.075 | 150 | 71.4 | 79.9 | 58.9 | 129 | 83.0 | 7.41 | 77.9 | 59.0 | 36.9 | 79.9 | 110 | 78.0 |
| **6** | 0.075 | 150 | 69.9 | 80.1 | 59.0 | 828 | 82.9 | 9.18 | 78.0 | 58.9 | 36.5 | 80.1 | 109 | 78.1 |
| **7** | 0.075 | 150 | 70.5 | 79.8 | 58.8 | 0.00 | 83.1 | 7.90 | 78.1 | 59.1 | 37.0 | 79.8 | 110 | 77.9 |
| **8** | 0.04 | 150 | 68.5 | 82.1 | 57.5 | 716 | 87.7 | 6.70 | 69.5 | 59.5 | 0.00 | 88.3 | 0.00 | 79.0 |
| **9** | 0.11 | 150 | 70.1 | 83.3 | 59.2 | 430 | 84.2 | 9.90 | 82.0 | 59.6 | 53.6 | 85.4 | 110 | 81.9 |
| **10** | 0.075 | 93 | 56.1 | 65.4 | 48.8 | 0.00 | 69.6 | 0.00 | 68.8 | 52.3 | 29.2 | 68.7 | 26.8 | 70.2 |
| **11** | 0.075 | 205 | 75.6 | 87.4 | 66.9 | 67.2 | 85.8 | 16.2 | 80.3 | 61.0 | 40.7 | 84.8 | 90.8 | 101 |
| **12** | 0.075 | 150 | 70.9 | 80.2 | 58.9 | 30.5 | 83.1 | 8.10 | 78.5 | 59.2 | 36.8 | 80.0 | 110 | 77.8 |
| **13** | 0.075 | 150 | 71.5 | 79.8 | 58.9 | 29.9 | 82.8 | 7.80 | 78.0 | 58.8 | 37.1 | 79.8 | 109 | 78.1 |
| **14** | 0.075 | 150 | 70.5 | 80.0 | 59.0 | 30.1 | 83.0 | 8.00 | 77.8 | 59.0 | 36.5 | 80.1 | 108 | 77.9 |

**Figure S3**: Response surface for percentage recoveries of metal ions using (5 M) HCl extracting reagent, as a function of temperature (TEMP), ^o^C and coal amount (CA), g at a constant extraction time of 5 min.

**Figure S4**: Response surface for percentage recoveries of metal ions using (2 M) HNO_3_ extracting reagent, as a function of temperature (TEMP), ^o^C and coal amount (CA), g at a constant extraction time of 5 min.

1. Corresponding author: E-mail address: nomvano.mketo@gmail.com or mketon@unisa.ac.za; Tel: +27114712032 [↑](#footnote-ref-2)
